# Deep Eutectic Solvents as Agents for Improving the Solubility of Edaravone: Experimental and Theoretical Considerations

**DOI:** 10.3390/molecules29061261

**Published:** 2024-03-12

**Authors:** Tomasz Jeliński, Maciej Przybyłek, Magdalena Mianowana, Kinga Misiak, Piotr Cysewski

**Affiliations:** Department of Physical Chemistry, Collegium Medicum of Bydgoszcz, Nicolaus Copernicus University in Toruń, Kurpińskiego 5, 85-096 Bydgoszcz, Poland; m.przybylek@cm.umk.pl (M.P.); 300804@stud.umk.pl (M.M.); 300805@stud.umk.pl (K.M.); piotrc@cm.umk.pl (P.C.)

**Keywords:** edaravone, solubility, Deep Eutectic Solvents, molecular complexes, cosolvency, choline chloride

## Abstract

In this study, both practical and theoretical aspects of the solubility of edaravone (EDA) in Deep Eutectic Solvents (DESs) were considered. The solubility of edaravone in some media, including water, can be limited, which creates the need for new efficient and environmentally safe solvents. The solubility of EDA was measured spectrophotometrically and the complex intermolecular interactions within the systems were studied with the COSMO-RS framework. Of the four studied DES systems, three outperformed the most efficient classical organic solvent, namely dichloromethane, with the DES comprising choline chloride and triethylene glycol, acting as hydrogen bond donor (HBD), in a 1:2 molar proportion yielding the highest solubility of EDA. Interestingly, the addition of a specific amount of water further increased EDA solubility. Theoretical analysis revealed that in pure water or solutions with high water content, EDA stacking is responsible for self-aggregation and lower solubility. On the other hand, the presence of HBDs leads to the formation of intermolecular clusters with EDA, reducing self-aggregation. However, in the presence of a stoichiometric amount of water, a three-molecular EDA–HBD–water complex is formed, which explains why water can also act as a co-solvent. The high probability of formation of this type of complexes is related to the high affinity of the components, which exceeds all other possible complexes.

## 1. Introduction

Edaravone (5-methyl-2-phenyl-4H-pyrazol-3-one, CAS Number: 89-25-8) is a novel antioxidant compound that exerts its effects through various mechanisms, including inhibiting non-enzymatic peroxidation and lipoxygenase activity, as well as preventing cerebral edema after ischemia [1,2]. It is also used in the treatment of amyotrophic lateral sclerosis (ALS) [3,4]. The above actions result from its antioxidant activity related to the fact that edaravone is an effective free radical scavenger [5,6]. In addition to its antioxidizing activity, edaravone also exhibits anti-inflammatory, anti-apoptotic, and anti-necrotic effects [7]. Interestingly, recent research has highlighted the potential nephroprotective effects of edaravone against various nephrotoxic agents [8,9]. Therefore, it is no surprise that edaravone attracts the interest of many scholars and is the subject of numerous studies [10,11]. A serious limitation in the application of this active pharmaceutical ingredient, resulting in poor bioavailability, is its low aqueous solubility, as evidenced by the categorization of edaravone as a class IV drug according to the Biopharmaceutics Classification System. The mentioned limited solubility of edaravone can be attributed to its chemical structure [12]. The presence of a phenyl group and a methyl group attached to the pyrazolone ring makes edaravone a relatively hydrophobic molecule, which is the reason for its decreased affinity for water, and thus low aqueous solubility. Also, the rather bulky structure of edaravone, and the lack of functional groups facilitating hydrogen bonding with water molecules may contribute to this limitation. Several solubility studies were conducted to find suitable solvents, which included both neat and binary systems [12,13,14,15]. The rather complicated tautomeric structure of edaravone was also investigated [16,17,18].

The importance of solubility studies for active pharmaceutical ingredients cannot be underestimated, as it is a crucial parameter influencing many aspects of a drug, including its reactivity, stability, and bioavailability. Therefore, it is not surprising that this property has been extensively investigated, especially from the perspective of the pharmaceutical industry [19,20,21,22,23,24]. Many efforts are undertaken to develop effective methods of solubility determination [25,26,27,28], as well as its improvement [29,30,31,32,33,34,35,36]. Also, the prediction of solubility is an important aspect of pharmaceutical practice, aiding in the selection of optimal solvents and their mixtures [37,38]. Particularly, the COSMO-RS framework [39] was proven to be a useful tool in the pharmaceutical industry not only for solubility modeling [40,41] but also for the description of two-phase systems [42,43] or determining co-crystallization abilities [44,45].

In this context, it must be emphasized that not all solvents or solubility-enhancement techniques are suitable and attractive for pharmaceutical applications. The main goal of current research, regardless of the field of application, is to provide solvents that are not only safe from the perspective of human health but also have a minimal environmental impact, i.e., the so-called “green solvents” [46,47,48,49]. This, of course, has become both a challenge and an opportunity for the pharmaceutical industry [50,51,52].

When the term “green solvents” appears, it can be immediately associated, among other things, with a specific group of designed solvents called Deep Eutectic Solvents, abbreviated as DESs [53,54,55]. Deep eutectic solvents differ from ionic liquids, even though they share many features, by the fact that they utilize non-ionic species as their components [56]. Also, very often, they include organic acids, alcohols, amino acids, sugars, or other plant-based primary metabolites [57,58]. DESs generally comprise a hydrogen bond acceptor (HBA) and a hydrogen bond donor (HBD). As the former, choline chloride is very often used due to its desirable properties, such as nontoxicity and low cost [59]. On the other hand, various polyols are commonly used as HBDs [60,61]. There are a number of properties that make DESs attractive as potential solubilizing media, including low volatility, sustainability and biodegradability, low cost and simplicity of preparation, and the ability to be fine-tuned for specific applications [62,63], although their environmental friendliness has been questioned [64]. The ecotoxicity of DESs is therefore a crucial aspect of their application, and several studies have evaluated their performance in this context, including the systems used in this work [65,66,67]. Despite these limitations, DESs are extensively utilized in many applications, including the pharmaceutical industry [68,69,70]. In fact, one of our previous studies was focused on the solubility of edaravone in DESs [71].

This particular study was aimed at determining the solubility of edaravone in a range of Deep Eutectic Solvents, thus extending our previous study and explaining the origins of the observed solubility increase in aqueous DES mixtures. The studied DESs comprised choline chloride as a hydrogen bond acceptor (HBA) and four different polyols playing the role of hydrogen bond donors (HBD). The theoretical analysis of the possible complexes occurring in the studied systems supplemented the experimental results. The presented results offer new solubility data for edaravone which can be utilized not only in future experiments but also for computational and modeling purposes. Also, the results can guide researchers in selecting deep eutectic systems for the effective dissolution of other active pharmaceutical ingredients. Finally, they allow for a better understanding of the observed phenomena occurring in the studied systems.

## 2. Results and Discussion

### 2.1. Solubility Determination

The current study is an extension of the previous investigations regarding the solubility of edaravone in organic solvents [72] and DES systems [71]. The main motivation is finding pharmaceutically acceptable systems with elevated solubility of this active pharmaceutical ingredient. In the former study, ethaline (ETA) and glyceline (GLE), i.e., deep eutectic systems involving choline chloride (ChCl) with ethylene glycol (EG) and glycerol (GL), were studied. Here, a similar reasoning was adopted, and four other choline chloride-based DES systems were investigated, which included 1,2-propanediol (P2D), 1,3-butanediol (B3D), diethylene glycol (DEG), and triethylene glycol (TEG) as HBD constituents. All of these selected compounds are quite often used in solubility studies as neat solvents, components of solvent mixtures, and constituents of Deep Eutectic Solvents [60,73,74,75,76,77,78,79].

In the initial phase of the solubility experiments, three distinct formulations of DESs were tested. These formulations encompassed an equal ratio of the two constituents, along with two- and four-times excess amounts of either of the four studied polyols. No formulations with an excessive amount of choline chloride were employed, as in previous experiences this was proven to be inefficient. In all four studied cases, the 1:2 molar ratio turned out to yield the highest solubility of edaravone, which is consistent with previous findings regarding ethylene glycol and glycerol. Also, for all systems in consideration, the 1:4 molar ratio proved to be the second most effective composition, with the unimolar proportion of both constituents being the least effective. In the case of P2D and B3D, the 1:4 molar ratio comprises about 80% of the optimal solubilization efficiency, while for the 1:1 proportion, this is about 60%. In the case of DEG and TEG, these values are around 85% and 70%, respectively. The application of TEG resulted in the highest solubility of edaravone among the studied systems, irrespective of the molar ratio. The solubility of edaravone at 298.15 K in the optimal system involving TEG was x_E_ = 0.2159 and was larger than for the eutectics studied previously [71]. Also, the deep eutectic solvent involving DEG was characterized by a greater solubility with x_E_ = 0.1769. On the other hand, systems with P2D and B3D performed worse (x_E_ = 0.0661 and x_E_ = 0.0760, respectively) than those utilized in the earlier study. Overall, the following decreasing order of solubilizing potential can be inferred (including systems from the earlier study): TEG > DEG > (ETA) > (GLY) > B3D > P2D. Interestingly, three of the four DESs studied here outperform dichloromethane, which has been recognized so far as the best organic solvent for edaravone dissolution (x_E_ = 0.0688) [14]. For TEG, DEG, and B3D, there is a 1.10-, 2.60-, and 3.14-fold respective increase in mole fraction solubility compared to this organic solvent. Only the P2D system is slightly less effective than dichloromethane. All the results discussed above are summarized in Table 1.

For the second phase of solubility experiments, the 1:2 molar ratio of ChCl:HBD was chosen as the one offering the highest increase in edaravone solubility. The systems in question were diluted with water and the dissolution effectiveness of the obtained ternary solvents composed of water, choline chloride, and one of four polyols was experimentally determined. For precise concentration profile monitoring, nine different DES-water compositions were considered, with a step of 0.1 mole fraction of water added to DES.

**Figure 1:**
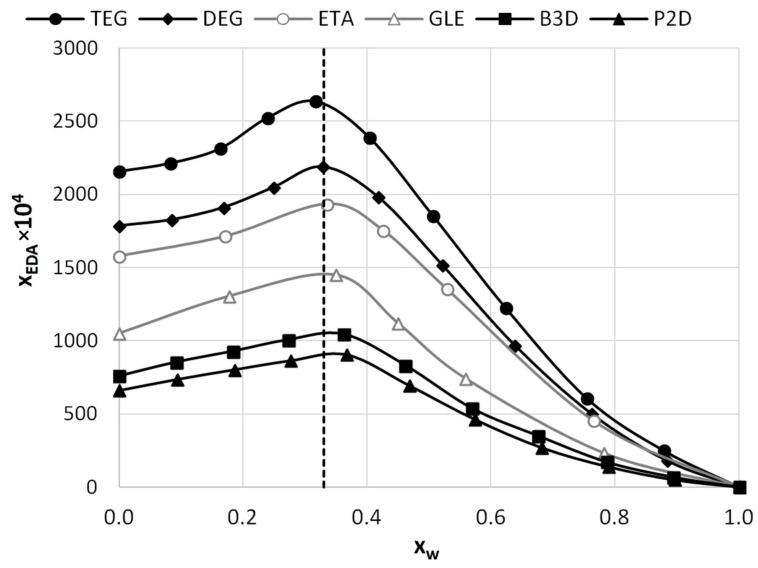
Mole fraction solubility of edaravone in mixtures of water and DESs comprising choline chloride and the second constituent in a 1:2 molar ratio. Grey lines and points denote the results obtained in a previous study [71]. On the abscissa, x_w_ represents the mole fractions of water in the saturated solutions. The dashed line corresponds to the unimolar proportion of HBD and water in saturated solutions.

In general, the discrepancy between the majority of API’s solubility in water and pure DESs is very high. Hence, water is supposed to be an extremely effective anti-solvent, which is also the case with EDA. However, it is known that in some cases the addition of water can improve the solubility of a particular active pharmaceutical ingredient compared to neat DESs [58,62,71,80], a phenomenon recognized in the literature as a co-solvency effect. Indeed, also here the application of DES-water mixtures resulted in the identification of the co-solvent role of water, contrary to its typically expected anti-solvent properties. The measured solubility profiles are collected in Table 2 and Figure 1, which also contain the results obtained in an earlier study. It is worth pointing out that the ordinate axis in Figure 1 represents the mole fraction of water in saturated systems. This way of presentation was chosen to emphasize the fact that, for all four studied eutectic systems, there is an unimolar proportion of HBD and water at maximum solubility. This, expressed in terms of solute-free solvent, corresponds to the values of x^*^_DES_ = 0.6 and x^*^_w_ = 0.4, where the subscript star stands for solute-free solutions. For the optimal DES-water mixture, the edaravone solubilities in P2D, B3D, DEG, and TEG systems are x_E_ = 0.0904, x_E_ = 0.1044, x_E_ = 0.2169, and x_E_ = 0.2635, respectively. These values correspond to an increase in edaravone solubility equal to around 1.37 times for P2D and B3D, as well as around 1.22 times for DEG and TEG. This shows a slight difference between these two pairs of studied systems, which is even visible in the shape of the solubility profiles. Obviously, it is an even greater solubility gain if compared to dichloromethane or other studied solvents [12,13,14,15]. It is worth noting that no known binary solvents, as well as no DES-water systems, studied earlier [71] performed better, and that the decreasing solubility order observed for neat eutectics remains valid also in the case of their mixtures with water.

It has to be emphasized that the solubility of edaravone in commonly known efficient organic solvents, such as DMSO [72] is significantly lower compared to that of considered DES. Additionally, conventional solvents cannot be used as drug excipients. This highlights the additional benefits offered by DESs, which are, in general, non-toxic systems suitable for pharmaceutical applications, including the development of liquid formulations. The recommended daily dosage of edaravone, namely 60 mg [81,82], is easily achievable with the studied systems, and their potential for fine-tuning is another promising aspect in formulating the new liquid forms of this drug. The separation of edaravone and the DES, after solubilization of the former, could be achieved by the addition of water, which plays an anti-solvent role at x_w_ > x_E_. This approach is widely utilized in the case of DESs [83,84] and allows for the recovery of both the solute (through precipitation) and the solvent (by evaporating the volatile anti-solvent).

### 2.2. Instrumental Characteristics of Studied Systems

**Figure 2:**
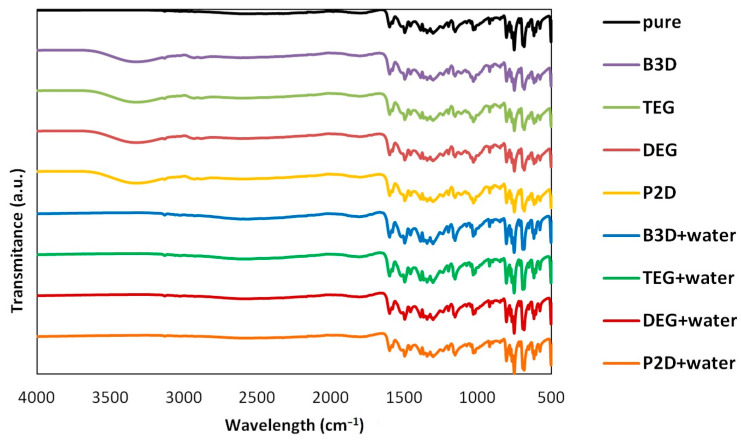
The FTIR spectra of edaravone residues after solubility measurements in neat DESs and DES–water mixtures (optimal composition, x^*^_DES_ = 0.6). The spectrum of pure edaravone is also provided.

**Figure 3:**
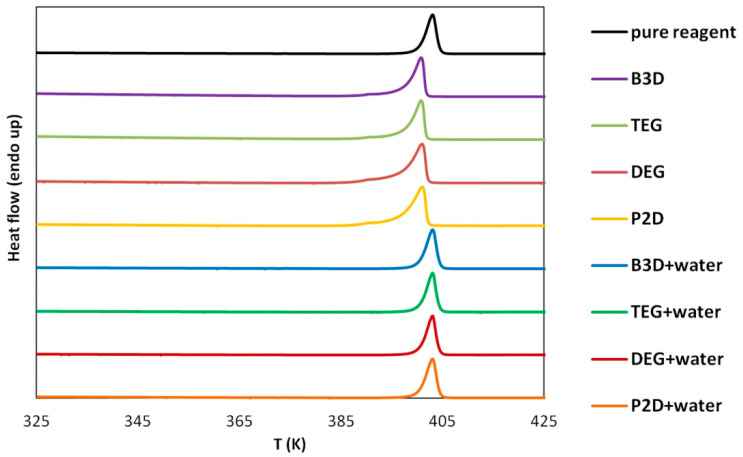
DSC curves of edaravone residues after solubility measurements in neat DESs and DES-water mixtures (optimal composition, x^*^_DES_ = 0.6). The DSC curve of pure edaravone is also provided.

To assess the possible impact of solvents on the crystalline structure of edaravone, the residues obtained after the post-shake flask procedure were subjected to analysis using DSC and FTIR–ATR techniques. An inherent difficulty in performing such experiments is the very low volatility of Deep Eutectic Solvents. While this property is often described as a “green feature” [85,86], and is promising from an environmental point of view, it also makes it difficult to properly dry the samples. Using very high temperatures in order to aid this process is also not recommended, as it might affect the crystalline structure of the sample. This problem was encountered during the previous studies involving edaravone in DESs [71]. In the case of each DES, two specific systems were studied, namely the pure eutectic in its optimal (1:2) molar ratio and the system containing water in its optimal amount (x^*^_DES_ = 0.6). Also, for comparison, the results obtained for pure edaravone are shown. The most important observation resulting from Figure 2 and Figure 3, depicting FTIR-ATR and DSC analysis, respectively, is the fact that the formation of solvates or new polymorphs was not observed for all studied systems. However, two classes of systems can be distinguished, and the differences can be attributed to the difficulties in drying the samples.

When taking into account the FTIR spectra, characteristic peaks of edaravone can be seen in all studied cases, as evidenced by comparison with the spectrum of pure edaravone. Nonetheless, for the pure DESs, characteristic peaks coming from alcohols can be seen, namely two low-intense bands at 2880 and 2936 cm^−1^ (υ_as_CH_2_ and υ_s_CH_2_) and one wide band at 3343 cm^−1^ (υOH), which is in accordance with the literature’s data [87]. As for the DSC analysis, the melting points (determined as onset values) of edaravone samples in DES–water mixtures are in very good accordance with the melting point of pure edaravone, which was determined as 400.73 K. For P2D + water, DEG + water, TEG + water, and B3D + water, the melting points are 400.79 K, 400.89 K, 400.91 K, and 400.75 K, respectively. Again, this is in good accordance with the literature [13,15]. For the pure eutectics however, a slight decrease in the melting point, resulting from impurities in the samples, can be observed and the values for neat P2D, DEG, TEG, and B3D are 396.82 K, 396.22 K, 397.31 K, and 396.93 K, respectively. The general picture inferred from the presented results is consistent with earlier findings [71].

### 2.3. Intermolecular Interactions of Edaravone in Studied DESs

**Figure 4:**
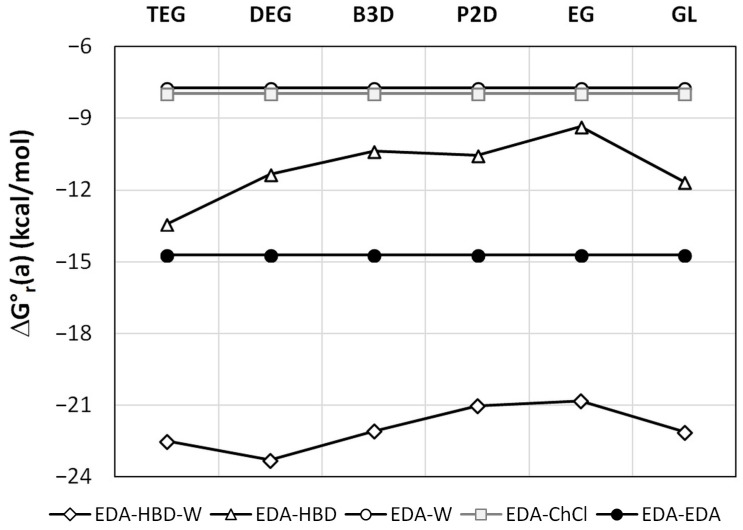
Edaravone self-affinity and affinity to DES components at room temperature. In the legend, the symbol HBD (hydrogen bond donor) denotes one of the components listed on the abscissa axis.

As it was already documented in Figure 1, the maximum solubility corresponds x_DES_^*^ = 0.6. Interestingly, this composition is quite close to a 1:1 molar ratio of HBD:water in either of the saturated systems. This intriguing observation can be rationalized by a detailed analysis of intermolecular interactions occurring in the saturated solutions of EDA in DESs. For this purpose, the most probable contacts were screened via a conformational analysis, as documented in the methodology section. The results are presented in Figure 4. The affinity values correspond to the activity equilibrium constants determined at a standard state (T = 298.15 K). Such values, by definition, are concentration-independent and can be used as a measure of interactions, irrespective of the mole fraction of components in a given DES. Hence, the affinities of EDA self-association and intermolecular pair formation with water and choline chloride are independent of the system. It is worth noting that the affinities of EDA to water and ChCl are nearly identical and equal to −7.7 and −8.0 kcal/mol, respectively. However, the self-association of EDA is much stronger, with ΔG°_r(a)_ = −14.7 kcal/mol. The other two types of clusters studied here comprise either hetero-molecular contacts involving an EDA–HBD pair or an EDA–HBD–W three-molecular complex. It is hypothesized that the presence of the latter clusters is responsible for the increased solubility of EDA in wet DESs. As directly inferred from Figure 4, such three-molecular complexes are very probable since the affinity for their formation is very high. Indeed, for all studied cases, the following two conditions are fulfilled, namely ΔG°_r(a)_^(EDA−HBD−W)^ < ΔG°_r(a)_^(EDA−HBD)^ and ΔG°_r(a)_^(EDA−HBD−W)^ < [ΔG°_r(a)_^(EDA−HBD)^ + ΔG°_r(a)_^(EDA−W)^]. This means that not only does the probability of EDA−HBD−W complex formation exceed the probability of EDA−HBD pairs occurrence, but there is also a non-negligible cooperative effect between EDA, HBD, and water. Thus, the increase in water concentration increases the solubility due to the rising concentration of the EDA−HBD−W cluster, stabilizing the dissolved form of this solute. However, after exceeding unimolar proportions, the excess amount of water acts as an anti-solvent by solvating HBD, which in turn enhances the EDA self-aggregation and eventual precipitation.

**Figure 5:**
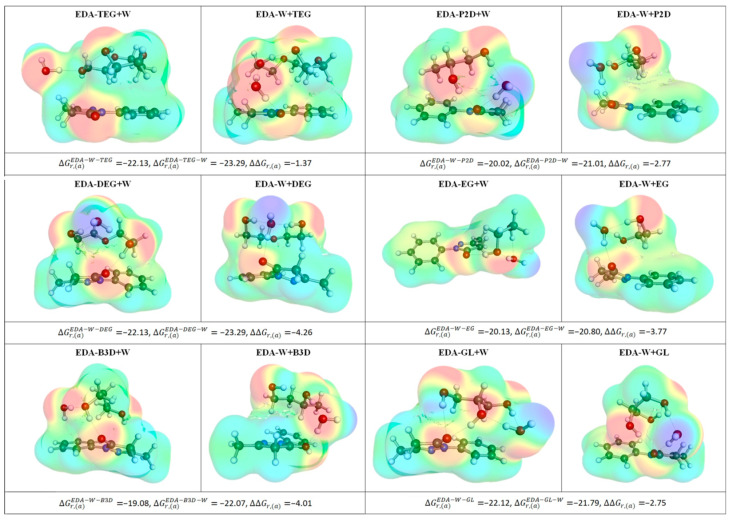
Illustration of the intermolecular complexes formed by EDA with HBD and water, where ΔΔG_r,(a)_ is a measure of cooperative effect computed as follows: ΔΔG_r,(a)_ = ΔG_r,(a)_^EDA−W−HBD^ − ΔG_r,(a)_^EDA−W^ − ΔG_r,(a)_^EDA−HBD^. All affinity energy values are provided in kcal/mol.

To further illustrate the clustering of EDA in the studied aqueous solutions of DESs, the most representative structures are collected in Figure 5, which comprises the results of two alternative minimization procedures, as described in the methodology section. It is evident that all clusters are stabilized by hydrogen bonding between EDA, water, and HBD, which is the reason for the high stability of all the considered complexes. Besides, the active role of the water molecule cannot be ignored, as it is involved in hydrogen-bonded bridges and is seldom bound via a single hydrogen bond.

## 3. Materials and Methods

### 3.1. Materials

Edaravone (EDA, CAS: 89-25-8, MW = 174.203 g/mol) with a purity of ≥99% was purchased from Sigma Aldrich (Saint Louis, MO, USA). The following DES constituents were also supplied by Sigma Aldrich and similarly had a purity of ≥99%: choline chloride (ChCl, CAS: 67-48-1), diethylene glycol (DEG, CAS: 111-46-6), triethylene glycol (TEG, CAS: 112-27-6), 1,2-propanediol (P2D, CAS: 57-55-6), and 1,3-butanediol (B3D, CAS: 107-88-0). Methanol (CAS: 67-56-1) supplied by Avantor Performance Materials (Gliwice, Poland) with a ≥99% purity was used throughout the study as a solvent. Choline chloride was dried before use, while all the other compounds were used without any initial procedures.

### 3.2. Preparation of the Calibration Curve

The initial step in creating a calibration curve required the preparation of an edaravone stock solution. This primary solution was then appropriately diluted with methanol in 10 mL volumetric flasks to produce solutions of decreasing concentrations. A total of ten solutions were prepared, with concentrations ranging from 0.0023 mg/mL to 0.023 mg/mL. These solutions were then analyzed spectrophotometrically. The wavelength corresponding to the highest absorbance was identified as 243 nm. Three separate curves were created, and the final curve was obtained by averaging the results. The linear regression equation was determined as A = 85.603 × C + 0.0179 (where A is absorbance, and C is concentration expressed in mg/mL), and the determination coefficient, R^2^, was calculated to be 0.999, indicating a high degree of accuracy. The limits of detection (LOD) and quantification (LOQ) were found to be 5.899 × 10^−4^ mg/mL and 1.770 × 10^−3^ mg/mL, respectively.

### 3.3. Preparation of the Samples and Solubility Measurements

In this study, the widely used shake flask method was employed to determine EDA solubility in considered DES systems. Importantly, this particular procedure has been applied in our previous reports and validated on various solutes and solvents [88,89,90,91]. The comparison of the obtained solubility values with the available literature data confirmed the reliability of this approach.

In all the investigated Deep Eutectic Solvents, choline chloride was constantly present as one of the constituents. The second component included one of the four compounds mentioned earlier, namely diethylene glycol (DEG), triethylene glycol (TEG), 1,2-propanediol (P2D), and 1,3-butanediol (B3D). To create a DES formulation, choline chloride was mixed with the second compound in various molar ratios within sealed test tubes. These tubes were then placed in a water bath at 363.15 K until a homogeneous solution was formed. The resulting DESs were used either in their pure form or in aqueous binary mixtures with varying proportions of water. After the solvents were prepared, excess amounts of edaravone (EDA) were added to the test tubes containing either neat DES or binary mixtures. In this manner, saturated solutions of EDA in the studied systems were obtained. The prepared samples were then placed in an Orbital Shaker Incubator ES-20/60 from Biosan (Riga, Latvia) and incubated for 24 h at 298.15 K. The temperature adjustment accuracy was maintained at 0.1 K, with a variation of ±0.5 K observed over a 24 h cycle. During the mixing process, all samples were agitated at a speed of 60 rev/min. Subsequently, the samples were filtered using a syringe equipped with a PTFE syringe filter featuring a pore size of 0.22 µm. To prevent precipitation, the test tubes, syringes, pipette tips, and filters were initially heated to match the temperature of the handled sample. Finally, small volumes of the filtered solution were transferred to test tubes containing methanol, and the diluted samples were measured on a spectrophotometer. Additionally, to determine the mole fractions of edaravone, the density of the samples was measured by weighing a fixed 1 mL volume of the solution in 10 mL volumetric flasks. Eppendorf (Hamburg, Germany) Reference 2 pipette was used throughout the study with a systematic error of 0.6 µL. The used RADWAG (Radom, Poland) AS 110 R2.PLUS analytical balance had a precision of 0.1 mg.

To determine the solubility of edaravone in the investigated systems, the samples prepared according to the aforementioned procedure were subjected to spectrophotometric analysis using an A360 spectrophotometer from AOE Instruments (Shanghai, China). The spectral data were recorded within the wavelength range of 190 nm to 500 nm, with a resolution of 1 nm. Initially, methanol was employed for spectrophotometer calibration, and it was also used to dilute the measured samples. Dilution was necessary to ensure that the absorbance values remained within the linearity threshold. Specifically, the absorbance values at 243 nm were considered, and based on the earlier prepared calibration curve, the concentration of edaravone in the samples was determined, along with its mole fractions. These values were derived by averaging the results obtained from three separate measurements.

### 3.4. Instrumental Analysis of the Samples

After the solubility measurements, the solid residues remaining in the test tubes were analyzed using Fourier transform infrared spectroscopy (FTIR) and differential scanning calorimetry (DSC). The FTIR spectra were obtained using a Spectrum Two spectrophotometer from Perkin Elmer (Waltham, MA, USA) equipped with an attenuated total reflection (ATR) device. The samples were analyzed within a wavenumber range of 450–4000 cm^−1^. For the DSC measurements, a DSC 6000 calorimeter from PerkinElmer (Waltham, MA, USA) was utilized. The heating rate was set at 10 K/min, and a nitrogen flow of 20 mL/min provided the inert atmosphere. The samples were placed in regular aluminum pans, and the instrument was calibrated using indium and zinc standards prior to the analysis.

### 3.5. Intermolecular Interactions Computations

**Scheme 1:**
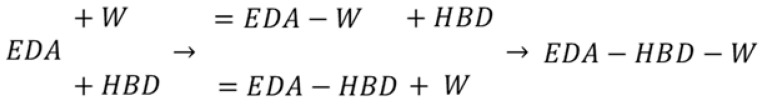
The schematic representation of EDA complexation with water or HBD (i.e., P2D, B3D, DEG, and TEG) in considered DES systems.

The solute–solute and solute–solvent intermolecular interactions are important characteristics of the solid–liquid equilibria (SLE) and were utilized in our precious works as sets of effective molecular descriptors for machine learning purposes [92,93]. Here, the mutual affinities of components in saturated solutions are determined to explain the role of water acting as a co-solvent in certain concentration ranges. The general computational procedure is analogous to our previous projects, so only indispensable details are provided here. The affinities of two- and three-molecular cluster formation are expressed as the negative values of the Gibbs free energies of corresponding reactions, as depicted in Scheme 1. Two paths leading to an EDA–HBD–W complex were considered, differing in the order of monomer attachments to EDA. Since both monomers and clusters are represented by a series of conformers, there are many combinations of possible products. For an adequate representation of the structural diversity of every contact, extended conformational analysis was performed for the identification of the lowest energy and the most probable complexes. This was done using the tandem of COSMOtherm (v.22.0.0) [94] and TURBOMOLE (v.7.5.1) [95] programs for cluster generation and full gradient geometry optimization, respectively. The former step was performed using the “CONTACT = {1 2} ssc_probability ssc_weak ssc_ang = 15.0” command for automatic generation of pairs by alteration of the mutual orientation of the two contacting molecules with a 15° step rotation interval. Not only hydrogen bonding but also weak interactions were included in the computation of the probability statistics. This step typically leads to a quite large number potential structures, the geometries of Ih are far from optimal. Hence, the second step involving structure optimization was performed using the RI-DFT BP86 (B88-VWN-P86) approach. The pool of obtained contacts underwent data reduction by excluding similar and high-energy geometries. Two criteria were used for this purpose, namely the RMSD values and the relative energy with respect to the most stable conformer. Hence, unique contacts within a 2.5 kcal/mol threshold window were included in the final pool of conformers representing a given complex. This procedure was applied to generate both two- and three-molecular complexes. It is worth mentioning that COSMOtherm, offering the description of reaction thermodynamics, requires two sets of conformers optimized in the gas phase and additionally in the bulk system, as defined by the COSMO-RS approach. Hence, the conformation analysis was repeated twice for these two environments. The final step required the generation of “cosmo” and “energy” files in the format suitable for application of the BP_TZVPD_FINE_21.ctd parameter set, i.e., the finest level available in COSMOtherm, which corresponds to computations on the RI-BP/TZVP//TZVPD-FINE level. The two alternative paths presented in Scheme 1 typically lead to different structures, and the final pool of conformers comprises the results of both ways of three-molecular complex formation. The actual affinity values were computed with COSMOtherm by using the option of concentration-independent Gibbs free energy, ΔG_r(a)_, for the reactions defined in Scheme 1. This value, accounting for the non-ideality of the system, guarantees that the affinity values are independent of concentrations of components in saturated EDA−DES systems.

## 4. Conclusions

The solubility of a particular active pharmaceutical ingredient is a very important factor from the point of view of the pharmaceutical industry. Here, both practical and theoretical aspects of the solubility of edaravone (EDA) in Deep Eutectic Solvents (DESs) were considered.

A number of DESs were investigated for their potential to improve the solubility of various chemical compounds. Indeed, three of the four DES systems studied were found to be more efficient than dichloromethane, which is the classical organic solvent best suited for dissolving EDA. The 1:2 molar ratio of the components of DESs, namely chlorine chloride and a selected polyol, was identified as the most efficient. The DES with triethylene glycol was the most effective, while the eutectic with diethylene glycol came second. These systems were also more efficient than the two systems with ethylene glycol and glycerol described in a previous study. It is known that the addition of a certain amount of water often improves the solubility of various compounds in DESs thanks to a cosolvency effect, and this was also the case in this study. An aqueous DES mixture of x_DES_^*^ = 0.6 and x_w_^*^ = 0.4 was responsible for the highest EDA solubility in all studied systems. Even though the increase in solubility was not very pronounced compared to pure DESs, it still outperformed all other binary solvents studied so far in the literature. Interestingly, the above composition of a DES-water mixture was found to be quite close to a 1:1 ratio of HBD:water, which inspired a detailed analysis of the intermolecular interactions in the saturated solutions of EDA in DESs to explain this phenomenon. In this context, the affinities of EDA to self-associate and form intermolecular complexes with water, choline chloride, and polyols (acting as HBDs) were studied. From the performed analysis, it was concluded that the three-molecular complex EDA-HBD-water is responsible for the increased solubility of EDA in aqueous DES mixtures. The formation of these complexes is very likely due to their high affinity, which exceeds the affinities of other complexes studied. Therefore, the addition of water to a given DES increases the concentration of the EDA-HBD-water complex, thus promoting the solubility of EDA in the system. However, if the water content exceeds an unimolar fraction, water acts as an antisolvent by solvating HBD and thus promoting the self-aggregation of EDA, which in turn leads to its precipitation. A detailed analysis of the three-molecule complexes of the studied systems revealed that their high stability is the result of hydrogen bonding between EDA, water, and HBD. Interestingly, water plays a crucial role in these complexes, as it is usually involved in multiple hydrogen bonds and not only in a single one, which is a factor stabilizing the considered complexes.

The presented findings provide novel solubility data for edaravone, applicable not just in forthcoming experiments but also for computational modeling. Additionally, the obtained results can assist researchers in choosing appropriate deep eutectic systems for enhancing the dissolution of various active pharmaceutical ingredients. Ultimately, they contribute to a deeper comprehension of the observed phenomena within the investigated systems.

## Figures and Tables

**Table 1:** The solubility of edaravone dissolved in DESs comprising choline chloride and the second constituent in different molar ratios (choline chloride first) at 298.15 K. Standard deviation values are given in parentheses.

HBD	1:1 ^1^		1:2		1:4	
	x_E_ (×10^4^)	c_E_ (mg/mL)	x_E_ (×10^4^)	c_E_ (mg/mL)	x_E_ (×10^4^)	c_E_ (mg/mL)
P2D	464.15 (±6.51)	79.01 (±1.00)	660.63 (±7.59)	120.6 (±1.27)	560.48 (±3.17)	110.86 (±0.52)
B3D	572.96 (±4.75)	88.53 (±0.74)	760.16 (±8.22)	123.21 (±1.25)	658.32 (±11.89)	112.47 (±2.01)
DEG	1142.86 (±12.68)	175.47 (±2.24)	1786.68 (±32.15)	274.56 (±4.47)	1430.41 (±33.48)	227.6 (±5.31)
TEG	1338.15 (±24.28)	182.36 (±3.13)	2158.65 (±28.83)	283.35 (±3.78)	1730.97 (±44.63)	224.62 (±5.19)

^1^ molar ratio of ChCl:HBD in non-aqueous solutions free of solute.

**Table 2:** The solubility of edaravone dissolved in mixtures of water and DESs comprising choline chloride and the second constituent in a 1:2 molar ratio at 298.15 K. Standard deviation values are given in parentheses.

x^*^_DES_ ^1^	P2D		B3D		DEG		TEG	
	x_E_ (×10^4^)	c_E_ (mg/mL)	x_E_ (×10^4^)	c_E_ (mg/mL)	x_E_ (×10^4^)	c_E_ (mg/mL)	x_E_ (×10^4^)	c_E_ (mg/mL)
0.0 ^2^	1.73 (±0.01)	1.72 (±0.01)	1.73 (±0.01)	1.72 (±0.01)	1.73 (±0.01)	1.72 (±0.01)	1.73 (±0.01)	1.72 (±0.01)
0.1	50.98 (±0.45)	34.08 (±0.32)	70.37 (±1.40)	44.66 (±0.84)	181.86 (±6.36)	108.49 (±3.61)	251.94 (±5.21)	136.67 (±2.81)
0.2	140.32 (±2.37)	71.00 (±1.19)	173.10 (±3.15)	81.10 (±1.32)	502.69 (±22.14)	210.35 (±7.66)	607.00 (±10.63)	217.30 (±3.64)
0.3	270.15 (±7.15)	108.69 (±2.67)	350.90 (±5.04)	127.93 (±1.52)	969.29 (±21.54)	305.56 (±5.60)	1223.75 (±19.47)	325.47 (±3.52)
0.4	463.56 (±8.70)	153.13 (±2.61)	538.01 (±9.72)	161.55 (±2.67)	1518.63 (±23.66)	384.60 (±4.36)	1852.86 (±13.62)	398.73 (±2.73)
0.5	694.74 (±26.13)	195.31 (±6.52)	831.86 (±8.04)	209.27 (±1.69)	1983.79 (±41.52)	431.02 (±7.05)	2388.18 (±23.29)	444.40 (±4.09)
0.6	904.23 (±9.14)	222.13 (±1.86)	1044.48 (±23.52)	229.98 (±4.43)	2189.62 (±40.44)	429.00 (±6.24)	2635.92 (±25.61)	447.50 (±3.27)
0.7	863.77 (±38.78)	195.04 (±7.79)	1007.49 (±15.00)	203.22 (±2.83)	2049.69 (±42.53)	377.45 (±6.40)	2524.97 (±37.72)	397.24 (±4.90)
0.8	801.94 (±10.87)	168.23 (±1.97)	928.82 (±34.85)	173.25 (±6.06)	1911.17 (±62.54)	331.84 (±9.52)	2313.80 (±40.79)	343.41 (±5.34)
0.9	736.17 (±10.62)	143.75 (±1.93)	854.66 (±13.27)	148.11 (±2.21)	1828.09 (±51.23)	299.48 (±7.79)	2213.70 (±45.66)	308.19 (±6.00)
1.0	660.63 (±7.59)	120.60 (±1.27)	760.16 (±8.22)	123.21 (±1.25)	1786.68 (±32.15)	274.56 (±4.47)	2158.65 (±28.83)	283.35 (±3.78)

^1^ x^*^_DES_ denotes the mole fractions of a particular DES in solute-free mixtures with water. ^2^ solubility of edaravone in water was obtained from a previous study [71].

## Data Availability

Data are contained within the article.

## References

[1] Nishi H., Watanabe T., Sakurai H., Yuki S., Ishibashi A. (1989). Effect of MCI-186 on brain edema in rats. Stroke.

[2] Abe K., Yuki S., Kogure K. (1988). Strong attenuation of ischemic and postischemic brain edema in rats by a novel free radical scavenger. Stroke.

[3] Drugbank Edaravone. https://go.drugbank.com/drugs/DB12243.

[4] Bhandari R., Kuhad A., Kuhad A. (2018). Edaravone: A new hope for deadly amyotrophic lateral sclerosis. Drugs Today.

[5] Mao Y.-F., Yan N., Xu H., Sun J.-H., Xiong Y.-C., Deng X.-M. (2009). Edaravone, a free radical scavenger, is effective on neuropathic pain in rats. Brain Res..

[6] Lin M., Katsumura Y., Hata K., Muroya Y., Nakagawa K. (2007). Pulse radiolysis study on free radical scavenger edaravone (3-methyl-1-phenyl-2-pyrazolin-5-one). J. Photochem. Photobiol. B Biol..

[7] Kikuchi K., Tancharoen S., Takeshige N., Yoshitomi M., Morioka M., Murai Y., Tanaka E. (2013). The Efficacy of Edaravone (Radicut), a Free Radical Scavenger, for Cardiovascular Disease. Int. J. Mol. Sci..

[8] Demir F., Demir M., Aygun H. (2020). Evaluation of the protective effect of edaravone on doxorubicin nephrotoxicity by [99mTc]DMSA renal scintigraphy and biochemical methods. Naunyn. Schmiedebergs. Arch. Pharmacol..

[9] Koike N., Sasaki A., Murakami T., Suzuki K. (2019). Effect of edaravone against cisplatin-induced chronic renal injury. Drug Chem. Toxicol..

[10] Fidalgo M., Ricardo Pires J., Viseu I., Magalhães P., Gregório H., Afreixo V., Gregório T. (2022). Edaravone for acute ischemic stroke–Systematic review with meta-analysis. Clin. Neurol. Neurosurg..

[11] Bailly C. (2019). Potential use of edaravone to reduce specific side effects of chemo-, radio- and immuno-therapy of cancers. Int. Immunopharmacol..

[12] Li Z. (2021). Equilibrium solubility of edaravone in some binary aqueous and non-aqueous solutions reconsidered: Extended Hildebrand solubility approach, transfer property and preferential solvation. J. Mol. Liq..

[13] Wu X., Yin X., Tang T., Zheng H., Xu W., Lin Z., Chen X., Li R., Zhao J., Han D. (2020). Solubility of Edaravone in Four Mixed Solvents at 273.15-313.15 K and Correlation of Jouyban-Acree and CNIBS/R-K Models. J. Chem. Eng. Data.

[14] Qiu J., Huang H., He H., Liu H., Hu S., Han J., Yi D., An M., Guo Y., Wang P. (2020). Solubility Determination and Thermodynamic Modeling of Edaravone in Different Solvent Systems and the Solvent Effect in Pure Solvents. J. Chem. Eng. Data.

[15] Li R., Yao L., Khan A., Zhao B., Wang D., Zhao J., Han D. (2019). Co-solvence phenomenon and thermodynamic properties of edaravone in pure and mixed solvents. J. Chem. Thermodyn..

[16] Abraham R.J., Cooper M.A., Aghamohammadi A., Afarinkia K., Liu X. (2022). The Use of MM/QM Calculations of 13C Chemical Shifts in the Analysis of Edaravone Tautomers. J. Solution Chem..

[17] Fakhraian H., Nafari Y. (2021). Preparative, mechanistic and tautomeric investigation of 1-phenyl and 1-methyl derivative of 3-methyl-5-pyrazolone. J. Chem. Sci..

[18] Freyer W., Köppel H., Radeglia R., Malewski G. (1983). ^1^H-NMR-, ^13^C-NMR-, and IR-Investigations Concerning Tautomerism of ^15^N-Labeled 3-Methyl-1-phenyl-Δ^2^-pyrazolin-5-one. J. Prakt. Chem..

[19] Martínez F., Jouyban A., Acree W.E. (2017). Pharmaceuticals solubility is still nowadays widely studied everywhere. Pharm. Sci..

[20] Savjani K.T., Gajjar A.K., Savjani J.K. (2012). Drug solubility: Importance and enhancement techniques. ISRN Pharm..

[21] Tran P., Pyo Y.-C., Kim D.-H., Lee S.-E., Kim J.-K., Park J.-S. (2019). Overview of the Manufacturing Methods of Solid Dispersion Technology for Improving the Solubility of Poorly Water-Soluble Drugs and Application to Anticancer Drugs. Pharmaceutics.

[22] Hancock B.C., York P., Rowe R.C. (1997). The use of solubility parameters in pharmaceutical dosage form design. Int. J. Pharm..

[23] Blagden N., de Matas M., Gavan P.T., York P. (2007). Crystal engineering of active pharmaceutical ingredients to improve solubility and dissolution rates. Adv. Drug Deliv. Rev..

[24] Khadka P., Ro J., Kim H., Kim I., Kim J.T., Kim H., Cho J.M., Yun G., Lee J. (2014). Pharmaceutical particle technologies: An approach to improve drug solubility, dissolution and bioavailability. Asian J. Pharm. Sci..

[25] Grossmann L., McClements D.J. (2023). Current insights into protein solubility: A review of its importance for alternative proteins. Food Hydrocoll..

[26] Sou T., Bergström C.A.S. (2018). Automated assays for thermodynamic (equilibrium) solubility determination. Drug Discov. Today Technol..

[27] Lu W., Chen H. (2022). Application of deep eutectic solvents (DESs) as trace level drug extractants and drug solubility enhancers: State-of-the-art, prospects and challenges. J. Mol. Liq..

[28] Suwanwong Y., Boonpangrak S. (2021). Molecularly imprinted polymers for the extraction and determination of water-soluble vitamins: A review from 2001 to 2020. Eur. Polym. J..

[29] Huang L., Tong W.-Q. (2004). Impact of solid state properties on developability assessment of drug candidates. Adv. Drug Deliv. Rev..

[30] Merisko-Liversidge E., Liversidge G.G. (2011). Nanosizing for oral and parenteral drug delivery: A perspective on formulating poorly-water soluble compounds using wet media milling technology. Adv. Drug Deliv. Rev..

[31] Scholz A., Abrahamsson B., Diebold S.M., Kostewicz E., Polentarutti B.I., Ungell A.-L., Dressman J.B. (2002). Influence of hydrodynamics and particle size on the absorption of felodipine in labradors. Pharm. Res..

[32] Janssens S., Van den Mooter G. (2009). Review: Physical chemistry of solid dispersions. J. Pharm. Pharmacol..

[33] Brewster M.E., Loftsson T. (2007). Cyclodextrins as pharmaceutical solubilizers. Adv. Drug Deliv. Rev..

[34] Korn C., Balbach S. (2014). Compound selection for development–Is salt formation the ultimate answer? Experiences with an extended concept of the “100mg approach”. Eur. J. Pharm. Sci..

[35] Chadha R., Bhalla Y., Vashisht M.K., Chadha K., Glebovsky V. (2015). Cocrystallization in Nutraceuticals. Recrystallization in Materials Processing.

[36] Seedher N., Kanojia M. (2009). Co-solvent solubilization of some poorly-soluble antidiabetic drugs. Pharm. Dev. Technol..

[37] Lovette M.A. (2022). Solubility Model to Guide Solvent Selection in Synthetic Process Development. Cryst. Growth Des..

[38] Modarresi H., Conte E., Abildskov J., Gani R., Crafts P. (2008). Model-Based Calculation of Solid Solubility for Solvent Selection—A Review. Ind. Eng. Chem. Res..

[39] Eckert F., Klamt A. (2002). Fast solvent screening via quantum chemistry: COSMO-RS approach. AIChE J..

[40] Przybyłek M., Miernicka A., Nowak M., Cysewski P. (2022). New Screening Protocol for Effective Green Solvents Selection of Benzamide, Salicylamide and Ethenzamide. Molecules.

[41] Wojeicchowski J.P., Ferreira A.M., Okura T., Pinheiro Rolemberg M., Mafra M.R., Coutinho J.A.P. (2022). Using COSMO-RS to Predict Hansen Solubility Parameters. Ind. Eng. Chem. Res..

[42] Buarque F.S., Lima T.S.P., Carniel A., Ribeiro B.D., Coelho M.A.Z., Souza R.L., Soares C.M.F., Pereira M.M., Lima Á.S. (2024). Hormones Concentration in an Aqueous Two-Phase System: Experimental and Computational Analysis. Chem. Eng. Technol..

[43] Buarque F.S., Lima N.S., Soares C.M.F., Marques M.N., Cavalcanti E.B., Pereira M.M., Souza R.L., Lima Á.S. (2021). Preconcentration and chromatographic detection of atrazine in real water sample using aqueous two-phase system based on tetrahydrofuran and glycerol. Environ. Qual. Manag..

[44] Guidetti M., Hilfiker R., Kuentz M., Bauer-Brandl A., Blatter F. (2023). Exploring the Cocrystal Landscape of Posaconazole by Combining High-Throughput Screening Experimentation with Computational Chemistry. Cryst. Growth Des..

[45] Przybyłek M., Ziółkowska D., Mroczyńska K., Cysewski P. (2017). Applicability of Phenolic Acids as Effective Enhancers of Cocrystal Solubility of Methylxanthines. Cryst. Growth Des..

[46] DeSimone J.M. (2002). Practical approaches to green solvents. Science.

[47] Jessop P.G. (2011). Searching for green solvents. Green Chem..

[48] Cvjetko Bubalo M., Vidović S., Radojčić Redovniković I., Jokić S. (2015). Green solvents for green technologies. J. Chem. Technol. Biotechnol..

[49] Häckl K., Kunz W. (2018). Some aspects of green solvents. Comptes Rendus Chim..

[50] e Silva A.P.S., Pires F.C.S., Ferreira M.C.R., Silva I.Q., Aires G.C.M., Ribeiro T.M., Ortiz E.G., Martins M.L.H.S., de Carvalho R.N., Innamiudin, Boddula R., Ahamed M.I., Asiri A.M. (2020). Case studies of green solvents in the pharmaceutical industry. Green Sustainable Process for Chemical and Environmental Engineering and Science, Solvents for the Pharmaceutical Industry.

[51] Becker J., Manske C., Randl S. (2022). Green chemistry and sustainability metrics in the pharmaceutical manufacturing sector. Curr. Opin. Green Sustain. Chem..

[52] Mishra M., Sharma M., Dubey R., Kumari P., Ranjan V., Pandey J. (2021). Green synthesis interventions of pharmaceutical industries for sustainable development. Curr. Res. Green Sustain. Chem..

[53] Santana-Mayor Á., Rodríguez-Ramos R., Herrera-Herrera A.V., Socas-Rodríguez B., Rodríguez-Delgado M.Á. (2021). Deep eutectic solvents. The new generation of green solvents in analytical chemistry. TrAC Trends Anal. Chem..

[54] Perna F.M., Vitale P., Capriati V. (2020). Deep eutectic solvents and their applications as green solvents. Curr. Opin. Green Sustain. Chem..

[55] Vanda H., Dai Y., Wilson E.G., Verpoorte R., Choi Y.H. (2018). Green solvents from ionic liquids and deep eutectic solvents to natural deep eutectic solvents. Comptes Rendus Chim..

[56] Zhang Q., De Oliveira Vigier K., Royer S., Jérôme F. (2012). Deep eutectic solvents: Syntheses, properties and applications. Chem. Soc. Rev..

[57] Choi Y.H., van Spronsen J., Dai Y., Verberne M., Hollmann F., Arends I.W.C.E., Witkamp G.-J., Verpoorte R. (2011). Are natural deep eutectic solvents the missing link in understanding cellular metabolism and physiology?. Plant Physiol..

[58] Dai Y., van Spronsen J., Witkamp G.-J., Verpoorte R., Choi Y.H. (2013). Natural deep eutectic solvents as new potential media for green technology. Anal. Chim. Acta.

[59] Jurić T., Uka D., Holló B.B., Jović B., Kordić B., Popović B.M. (2021). Comprehensive physicochemical evaluation of choline chloride-based natural deep eutectic solvents. J. Mol. Liq..

[60] Wang H., Liu S., Zhao Y., Wang J., Yu Z. (2019). Insights into the Hydrogen Bond Interactions in Deep Eutectic Solvents Composed of Choline Chloride and Polyols. ACS Sustain. Chem. Eng..

[61] Biernacki K., Souza H.K.S., Almeida C.M.R., Magalhães A.L., Goncąlves M.P. (2020). Physicochemical Properties of Choline Chloride-Based Deep Eutectic Solvents with Polyols: An Experimental and Theoretical Investigation. ACS Sustain. Chem. Eng..

[62] Espino M., de los Ángeles Fernández M., Gomez F.J.V., Silva M.F. (2016). Natural designer solvents for greening analytical chemistry. TrAC Trends Anal. Chem..

[63] Paiva A., Craveiro R., Aroso I., Martins M., Reis R.L., Duarte A.R.C. (2014). Natural Deep Eutectic Solvents–Solvents for the 21st Century. ACS Sustain. Chem. Eng..

[64] Lomba L., Ribate M.P., Zaragoza E., Concha J., Garralaga M.P., Errazquin D., García C.B., Giner B. (2021). Deep Eutectic Solvents: Are They Safe?. Appl. Sci..

[65] De Morais P., Gonçalves F., Coutinho J.A.P., Ventura S.P.M. (2015). Ecotoxicity of Cholinium-Based Deep Eutectic Solvents. ACS Sustain. Chem. Eng..

[66] Macário I.P.E., Jesus F., Pereira J.L., Ventura S.P.M., Gonçalves A.M.M., Coutinho J.A.P., Gonçalves F.J.M. (2018). Unraveling the ecotoxicity of deep eutectic solvents using the mixture toxicity theory. Chemosphere.

[67] Lapeña D., Errazquin D., Lomba L., Lafuente C., Giner B. (2021). Ecotoxicity and biodegradability of pure and aqueous mixtures of deep eutectic solvents: Glyceline, ethaline, and reline. Environ. Sci. Pollut. Res..

[68] Emami S., Shayanfar A. (2020). Deep eutectic solvents for pharmaceutical formulation and drug delivery applications. Pharm. Dev. Technol..

[69] Hikmawanti N.P.E., Ramadon D., Jantan I., Mun’im A. (2021). Natural Deep Eutectic Solvents (NADES): Phytochemical Extraction Performance Enhancer for Pharmaceutical and Nutraceutical Product Development. Plants.

[70] Liu Y., Wu Y., Liu J., Wang W., Yang Q., Yang G. (2022). Deep eutectic solvents: Recent advances in fabrication approaches and pharmaceutical applications. Int. J. Pharm..

[71] Cysewski P., Jeliński T., Przybyłek M. (2023). Intermolecular Interactions of Edaravone in Aqueous Solutions of Ethaline and Glyceline Inferred from Experiments and Quantum Chemistry Computations. Molecules.

[72] Przybyłek M., Jeliński T., Mianowana M., Misiak K., Cysewski P. (2023). Exploring the Solubility Limits of Edaravone in Neat Solvents and Binary Mixtures: Experimental and Machine Learning Study. Molecules.

[73] Esfahani H.S., Khoshsima A., Pazuki G. (2020). Choline chloride-based deep eutectic solvents as green extractant for the efficient extraction of 1-butanol or 2-butanol from azeotropic n-heptane + butanol mixtures. J. Mol. Liq..

[74] Chen Q., He N., Fan J., Song F. (2022). Physical Properties of Betaine-1,2-Propanediol-Based Deep Eutectic Solvents. Polymers.

[75] Bu F., Zhao Y., Li B., Zhang X., Li J. (2023). The effect of choline chloride-butanediol based deep eutectic solvents on ultrasound-assisted extraction, antioxidant activity and stability of anthocyanins extracted from Perilla frutescens (L.) Britt. Sustain. Chem. Pharm..

[76] Basaiahgari A., Panda S., Gardas R.L. (2018). Effect of Ethylene, Diethylene, and Triethylene Glycols and Glycerol on the Physicochemical Properties and Phase Behavior of Benzyltrimethyl and Benzyltributylammonium Chloride Based Deep Eutectic Solvents at 283.15–343.15 K. J. Chem. Eng. Data.

[77] Gabriele F., Chiarini M., Germani R., Tiecco M., Spreti N. (2019). Effect of water addition on choline chloride/glycol deep eutectic solvents: Characterization of their structural and physicochemical properties. J. Mol. Liq..

[78] Aissaoui T., Benguerba Y., AlNashef I.M. (2017). Theoretical investigation on the microstructure of triethylene glycol based deep eutectic solvents: COSMO-RS and TURBOMOLE prediction. J. Mol. Struct..

[79] Hayyan M., Aissaoui T., Hashim M.A., AlSaadi M.A.H., Hayyan A. (2015). Triethylene glycol based deep eutectic solvents and their physical properties. J. Taiwan Inst. Chem. Eng..

[80] Cysewski P., Jeliński T., Cymerman P., Przybyłek M. (2021). Solvent Screening for Solubility Enhancement of Theophylline in Neat, Binary and Ternary NADES Solvents: New Measurements and Ensemble Machine Learning. Int. J. Mol. Sci..

[81] Nakamaru Y., Kinoshita S., Kawaguchi A., Takei K., Palumbo J., Suzuki M. (2017). Pharmacokinetic profile of edaravone: A comparison between Japanese and Caucasian populations. Amyotroph. Lateral Scler. Front. Degener..

[82] Cho H., Shukla S. (2020). Role of Edaravone as a Treatment Option for Patients with Amyotrophic Lateral Sclerosis. Pharmaceuticals.

[83] Isci A., Kaltschmitt M. (2022). Recovery and recycling of deep eutectic solvents in biomass conversions: A review. Biomass Convers. Biorefinery.

[84] Nguyen H.V.D., De Vries R., Stoyanov S.D. (2020). Natural Deep Eutectics as a “green” Cellulose Cosolvent. ACS Sustain. Chem. Eng..

[85] Ratti R. (2020). Industrial applications of green chemistry: Status, Challenges and Prospects. SN Appl. Sci..

[86] Zhao H., Xia S., Ma P. (2005). Use of ionic liquids as ‘green’ solvents for extractions. J. Chem. Technol. Biotechnol..

[87] Salehpour S., Dubé M.A. (2012). Reaction Monitoring of Glycerol Step-Growth Polymerization Using ATR-FTIR Spectroscopy. Macromol. React. Eng..

[88] Cysewski P., Jeliński T., Przybyłek M. (2022). Application of COSMO-RS-DARE as a Tool for Testing Consistency of Solubility Data: Case of Coumarin in Neat Alcohols. Molecules.

[89] Przybyłek M., Kowalska A., Tymorek N., Dziaman T., Cysewski P. (2021). Thermodynamic Characteristics of Phenacetin in Solid State and Saturated Solutions in Several Neat and Binary Solvents. Molecules.

[90] Cysewski P., Przybyłek M., Kowalska A., Tymorek N. (2021). Thermodynamics and Intermolecular Interactions of Nicotinamide in Neat and Binary Solutions: Experimental Measurements and COSMO-RS Concentration Dependent Reactions Investigations. Int. J. Mol. Sci..

[91] Cysewski P., Przybyłek M., Rozalski R. (2021). Experimental and theoretical screening for green solvents improving sulfamethizole solubility. Materials.

[92] Cysewski P., Jeliński T., Przybyłek M., Nowak W., Olczak M. (2022). Solubility Characteristics of Acetaminophen and Phenacetin in Binary Mixtures of Aqueous Organic Solvents: Experimental and Deep Machine Learning Screening of Green Dissolution Media. Pharmaceutics.

[93] Cysewski P., Przybyłek M., Jeliński T. (2023). Intermolecular Interactions as a Measure of Dapsone Solubility in Neat Solvents and Binary Solvent Mixtures. Materials.

[94] Dassault Systèmes, Biovia (2022). COSMOtherm.

[95] TURBOMOLE GmbH (2021). TURBOMOLE.

